# Supplementary figures and images for: Genetic deletion of Autotaxin from CD11b+ cells decreases the severity of experimental autoimmune encephalomyelitis
Source: PLoS One. 2020 Apr 2;15(4):e0226050. doi: 10.1371/journal.pone.0226050 (PMC7117669; doi:10.1371/journal.pone.0226050)

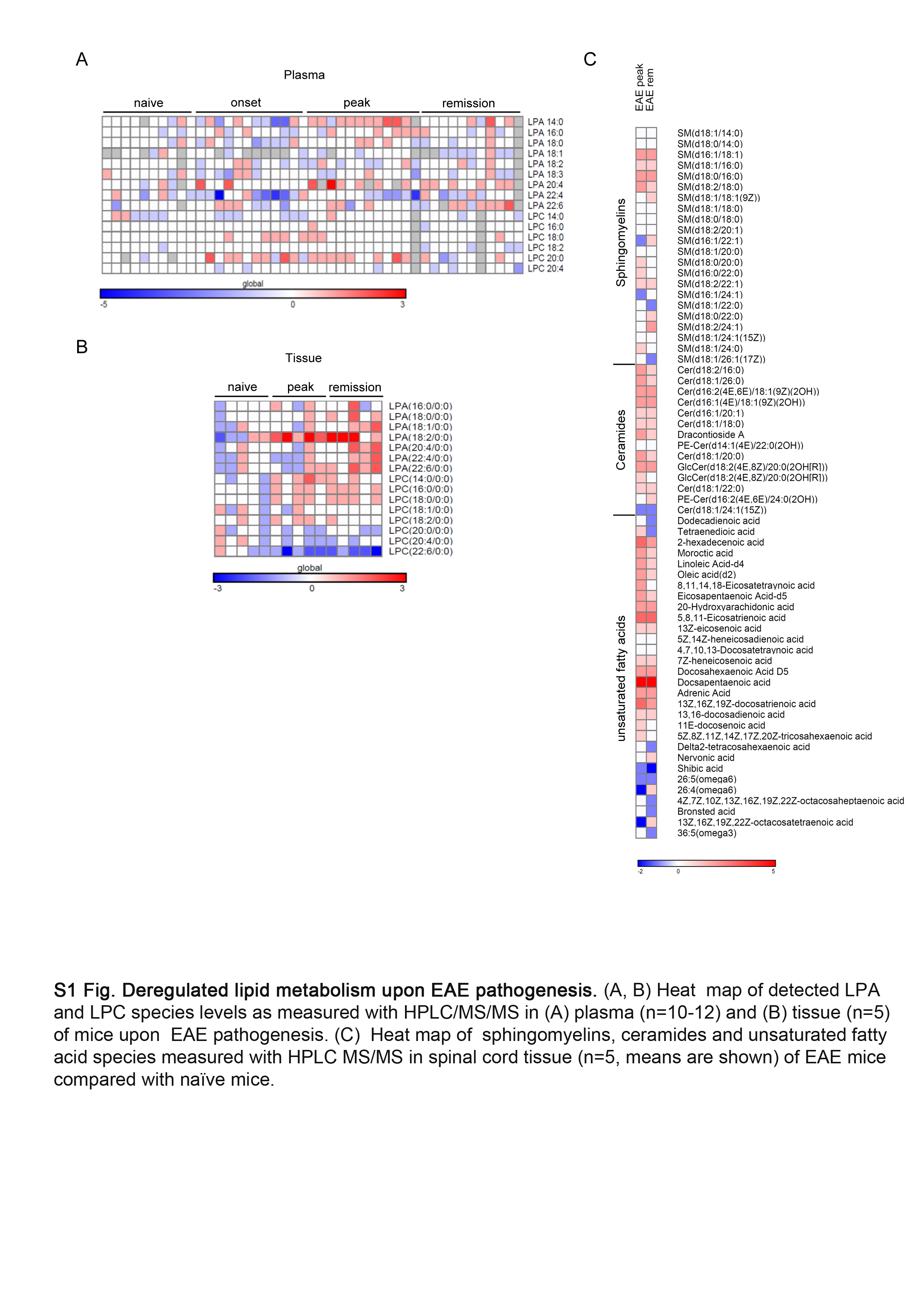

Supplement: S1 Fig — (A, B) Heat map of detected LPA and LPC species levels as measured with HPLC/MS/MS in (A) plasma (n = 10–12) and (B) tissue (n = 5) of mice upon EAE pathogenesis. (C) Heat map of sphingomyelins, ceramides and unsaturated fatty acid species measured with HPLC MS/MS in spinal cord tissue (n = 5, means are shown) of EAE mice compared with naïve mice. (TIF) [file pone.0226050.s001.tif]

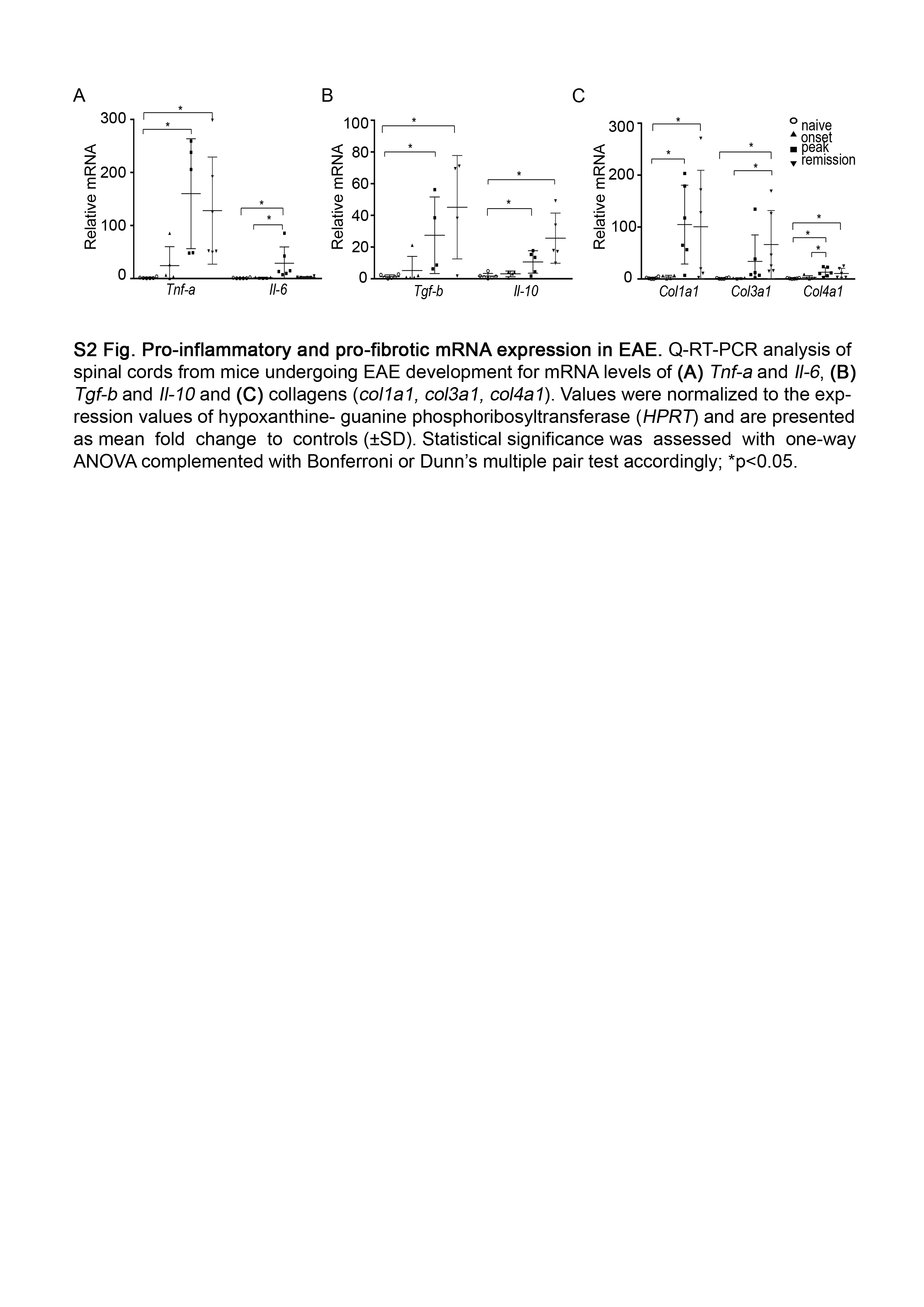

Supplement: S2 Fig — Q-RTPCR analysis of spinal cords from mice undergoing EAE development for mRNA levels of (A) Tnf-a and Il-6, (B) Tgf-b and Il-10 and (C) collagens (col1a1, col3a1, col4a1). Values were normalized to the expression values of hypoxanthine-guanine phosphoribosyltransferase (HPRT) and are presented as mean fold change to controls (±SD). Statistical significance was assessed with one-way ANOVA complemented with Bonferroni or Dunn’s multiple pair test accordingly; *p<0.05. (TIF) [file pone.0226050.s002.tif]

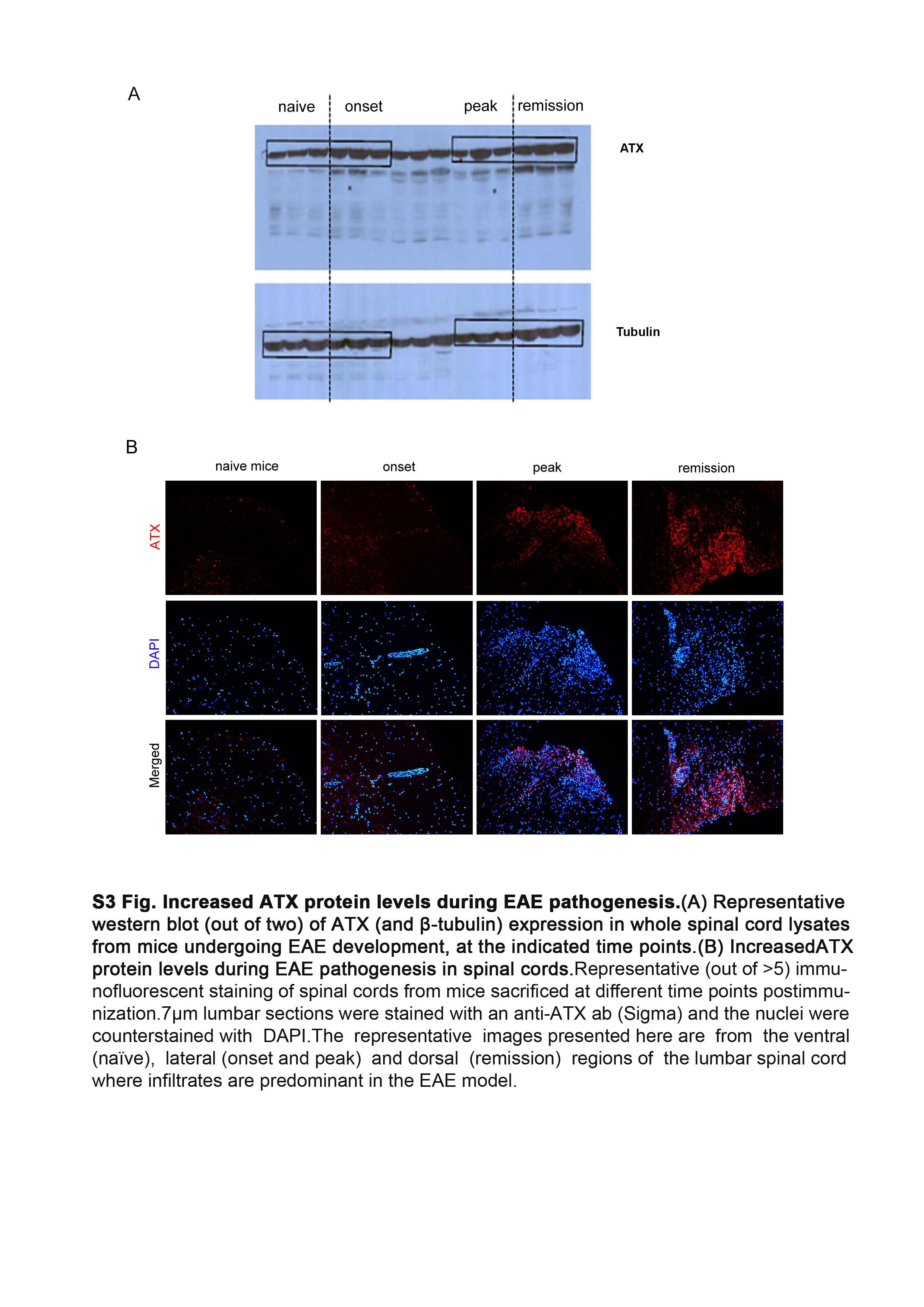

Supplement: S3 Fig — (A) Representative western blot (out of two) of ATX (and β-tubulin) expression in whole spinal cord lysates from mice undergoing EAE development, at the indicated time points. (B) Increased ATX protein levels during EAE pathogenesis in spinal cords. Representative (out of >5) immunofluorescent staining of spinal cords from mice sacrificed at different time points post immunization. 7μm lumbar sections were stained with an anti-ATX ab (Sigma) and the nuclei were counterstained with DAPI. The representative images presented here are from the ventral (naïve), lateral (onset and peak) and dorsal (remission) regions of the lumbar spinal cord, where infiltrates are predominant in the EAE model. (TIF) [file pone.0226050.s003.tif]

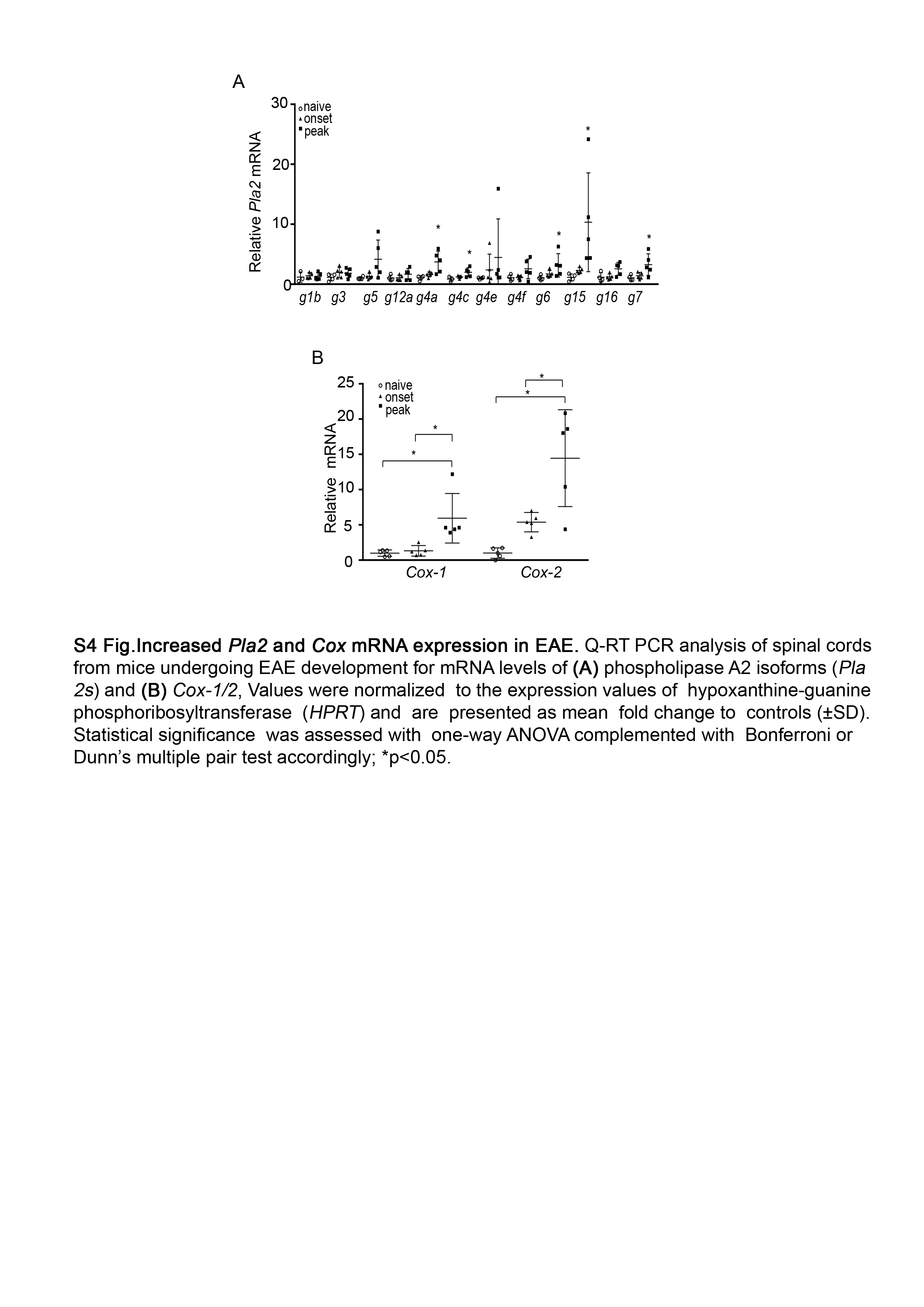

Supplement: S4 Fig — Q-RTPCR analysis of spinal cords from mice undergoing EAE development for mRNA levels of (A) phospholipase A2 isoforms (Pla2s) and (B) Cox-1/2. Values were normalized to the expression values of hypoxanthine-guanine phosphoribosyltransferase (HPRT) and are presented as mean fold change to controls (±SD). Statistical significance was assessed with one-way ANOVA complemented with Bonferroni or Dunn’s multiple pair test accordingly; *p<0.05. (TIF) [file pone.0226050.s004.tif]
