# Supplementary material for: Genetic deletion of Autotaxin from CD11b+ cells decreases the severity of experimental autoimmune encephalomyelitis
Source: PLoS One. 2020 Apr 2;15(4):e0226050. doi: 10.1371/journal.pone.0226050 (PMC7117669; doi:10.1371/journal.pone.0226050)
Supplement: S1 Table — (PDF) [file pone.0226050.s005.pdf]

Supplemental Table 1. Q-RT- PCR primers

Gene		Primer sequence (5′-3′)	Product (bp)
Hprt	s<br>as	GGCCAGACTTTGTTGGATTT<br>CAGATTCAACTTGCGCTCAT	154
Enpp2	s<br>as	GATGCATTCCTTGTAACCAACA<br>TCATCCTCAATGTCACGTAAGC	173
Enpp2	s<br>as	GTGAAATATTCTTAATGCCTCTCTG<br>GCCTTCCACATACTGTTTAATTCC	410
Enpp2-γ	s<br>as	GAAACCGGAAAATTCAGAGG<br>CACTTTCAAAGTCCGTATGG	154
Il-6	s<br>as	TAGTCCTTCCTACCCCAATTTCC<br>TTGGTCCTTAGCCACTCCTTC	335
Il-10	s<br>as	GCTCCTAGAGCTGCGGACT<br>TGTTGTCCAGCTGGTCCTTT	199
Tnf-a	s<br>as	CCTGTAGCCCACGTCGTAG<br>GGGAGTAGACAAGGTACAACCC	148
Tgf-b	s<br>as	CTCCCGTGGCTTCTAGTGC<br>GCCTTAGTTTGGACAGGATCTG	133
Col1a1	s<br>as	CTACTACCGGGCCGATGATG<br>CGATCCAGTACTCTCCGCTC	188
Col3a1	s<br>as	GCCCACAGCCTTCTACAC<br>CCAGGGTCACCATTTCTC	73
Col4a1	s<br>as	CAGGTGTGCGGTTTGTGAAG<br>TGGTGTGCATCACGAAGGAA	125
Lpar1	s<br>as	GAGGAATCGGGACACCATGAT<br>TGAAGGTGGCGCTCATCT	227
Lpar2	s<br>as	GACCACACTCAGCCTAGTCAAGAC<br>CAGCATCTCGGCAGGAAT	200
Lpar3	s<br>as	GCTCCCATGAAGCTAATGAAGACA<br>TACGAGTAGATGATGGGG	188
Lpar4	s<br>as	AGTGCCTCCCTGTTTGTCTTC<br>GCCAGTGGCGATTAAAGTTGTAA	142
Lpar5	s<br>as	ACCCTGGAGGTGAAAGTC<br>GACCACCATATGCAAACG	176
Lpar6	s<br>as	GATCACTCTCTGCATCGCTGTTTC<br>CCCTGAACTTCAGAGAACCTGGAG	141
Pla2g1b	s<br>as	CACCCCAGTGGACGACTTAG<br>GCATTTGTTGTTTTTGGCGCT	169
Pla2g3	s<br>as	AGAGACCACAGGGCCATTAAG<br>GCTGTAGAATGACATGGTGCT	141
Pla2g6	s<br>as	GCAAGCTGATTACCAGGAAGG<br>GAGAGAAGAGGGGGTGAGTTG	129
Pla2g12a	s<br>as	GCAACGGCATCCACAAGATAG<br>CATAGCGTGGAACAGGCTTC	117
Pla2g4a	s<br>as	CAGCACATTATAGTGGAACACCA<br>AGTGTCCAGCATATCGCCAAA	102
Pla2g4c	s<br>as	TGGCTGGGAATCCTGGGAA<br>GAGAGCACAGGTGGTGAGTC	185
Pla2g4E	s<br>as	ATGGTGACAGACTCCTTCGAG<br>CCTCTGCGTAAAGCTGTGG	118
Pla2g4f	s<br>as	AGCCATACTGCTACGGAAGAC<br>TTTGGACAACTTATCTGTGTGCT	136
Pla2g16	s<br>as	GGACCCAAGCAAAGGCATCC<br>CCAGCTCCTGCGATTTCACT	200
Pla2g7	s<br>as	CTTTTCACTGGCAAGACACATCT<br>CGACGGGGTACGATCCATTTC	132
Plpp1	s<br>as	TGTACTGCATGCTGTTTGTCGCAC<br>TGACGTCACTCCAGTGGTGTTTGT	159
Plpp2	s<br>as	TCCTTTGGCATGTATTGCATGT<br>AAGGCCACCAAGAAGAACTGA	110
Plpp3	s<br>as	ATAAACGATGCTGTGCTCTGTGCG<br>TTTGCTGTCTTCTCCTCTGCACCT	318

S: sense; as: antisense; bp: base pairs
